# Editorial: Advance in Molecularly Imprinted Polymers

**DOI:** 10.3390/polym15153199

**Published:** 2023-07-27

**Authors:** Michał Cegłowski

**Affiliations:** Faculty of Chemistry, Adam Mickiewicz University, Uniwersytetu Poznańskiego 8, 61-614 Poznan, Poland; michal.ceglowski@amu.edu.pl

Molecularly imprinted polymers (MIPs), due to their unique recognition properties, have found various applications, mainly in extraction and separation techniques; however, their implementation in other research areas, such as sensor construction and drug delivery, has also been substantial. These advances could not be achieved without developing new polymers and monomers that can be successfully applied in MIP synthesis and improve the range of their applications. Although much progress has been made in MIP development, more investigation must be conducted to obtain materials that can fully deserve the name of artificial antibodies.

The “Advance in Molecularly Imprinted Polymers” Special Issue connects original research papers and reviews presenting recent advances in the design, synthesis, and broad applications of molecularly imprinted polymers. The Special Issue content covers various topics related to MIP chemistry, which include their molecular modeling, application of new monomers and synthesis techniques for MIP preparation, synthesis, and application of ion-imprinted materials, selective extraction of organic molecules, sensor preparation, and MIP applications in medicine. The presented collection of scientific papers shows that research in MIP chemistry is diversified, ultimately improving the desired properties and creating new potential applications for these materials.

Bivián-Castro et al. [1] obtained a Cu(II)-imprinted polymer using a phenanthroline–divinylbenzoate complex as a functional monomer and ethylene glycol dimethacrylate as a cross-linker. The Cu(II)-loaded MIP appeared to be a mesoporous material with a pore diameter of around 4 nm. The Cu(II)-unloaded ion-imprinted polymer was considered a microporous material, as the pore diameters were less than 2 nm. The maximum adsorption capacity of the ion imprinted polymer was 287.45 mg g^−1^. The findings of this study indicate the potential of the obtained ion-imprinted polymers for the selective extraction of heavy metal ions from polluted waters.

Ion-imprinted polymers were studied by Kondaurov et al. [2]. They obtained molecularly imprinted polymers based on methacrylic acid and 4-vinylpyridine that were selective towards samarium and gadolinium ions. The obtained data showed that the sorption degree of samarium and gadolinium ions by the obtained MIPs reached almost 90%. The additional benefit of the MIPs was the fact that simultaneous sorption of accompanying metal ions was not observed. The results suggested that these MIPs could be highly effective alternatives to the existing sorption technologies.

Zaharia et al. [3] obtained ligand-free nanogels that were molecularly imprinted with bee venom-derived phospholipase A2 (PLA2) enzyme. This enzyme acts synergistically with the polyvalent cations in the venom, creating an increased hemolytic effect and quick access of toxins into the bloodstream. The research aim was to obtain nanogel materials capable of binding PLA2, which would could remove PLA2 proteins from the bite zone, reducing the venom’s toxicity. The rebinding experiments proved the specificity of the molecularly imprinted ligand-free nanogels for PLA2, with rebinding capacities up to 8-fold higher than the reference non-imprinted nanogel. The in vitro cell viability experiments showed the potential of the obtained ligand-free nanogels and suggested that they can be used for future therapies against bee envenomation.

Megahed et al. [4] obtained MIPs that can be used for the selective extraction of quinic acid from coffee bean extract. The authors used computational modeling to optimize the process of MIP preparation. It was used to calculate the optimum ratio for allylamine, methacrylic acid, and 4-vinylpyridine, which were used as functional monomers. The application of these MIP materials to extract quinic acid from aqueous coffee extract showed a high recovery, reaching 82%. The developed procedure is promising for the selective extraction of quinic acid from different complex herbal extracts and may be scaled to industrial applications.

Six aromatic *N*-(2-arylethyl)-2-methylprop-2-enamides with various substituents in the benzene ring were obtained by Sobiech et al. [5] using 2-arylethylamines and methacryloyl chloride as the primary reagents. The obtained compounds were used for covalent imprinting in the synthesis of molecularly imprinted polymers, followed by the hydrolysis of an amide linkage. The adsorption studies proved a high affinity towards tyramine and L-norepinephrine, which were used as target biomolecules. This method proves the applicability of covalent imprinting with the use of tailor-made monomers.

Piletsky et al. [6] presented the application of iodo silanes and compared it to commonly used amino silanes for the solid-phase synthesis of MIP nanoparticles. The silanes were used for peptide immobilization, and the obtained MIPs were specific toward an epitope of the epidermal growth factor receptor. The results showed that both silanes produced MIPs with excellent affinity; nevertheless, for the iodo silanes, fewer experimental steps were needed and the procedure required less expensive reagents.

The synthesis of 5-fluorouracil-imprinted microparticles and their application in prolonged drug delivery was reported by Cegłowski et al. [7]. The authors prepared MIPs using two different cross-linkers: ethylene glycol dimethacrylate and trimethylolpropane trimethacrylate. The calculated highest cumulative release was highly dependent on the cross-linker applied during the synthesis. The overall cumulative release was much higher for trimethylolpropane trimethacrylate-based MIPs. The highest cumulative release was obtained at pH 7.4 and the lowest at pH 2.2. As a result, it was demonstrated that the selection of the cross-linker should be considered during the design of materials used for drug delivery.

Jumadilov et al. [8] studied the effectiveness of the application of intergel systems and MIPs for the selective sorption and separation of neodymium and scandium ions. The intergel system method was proven cheaper and easier in this application; however, some accompanying sorption of another metal from the model solution was observed. On the other hand, the method based on MIPs was more expensive but showed higher sorption properties. The obtained results can be successfully applied to upgrade the existing sorption technologies.

Chien et al. [9] developed a fluorescent probe for specific biorecognition by a facile method in which amphiphilic random copolymers were encapsulated with hydrophobic upconversion nanoparticles. The self-folding ability of the amphiphilic copolymers allowed the formation of MIPs with template-shaped cavities that were selective towards albumin and hemoglobin. The results showed that the fluorescence was quenched when hemoglobin was adsorbed on the fluorescent probes. This effect was not observed for albumin. These fluorescent probes have the potential to be applied for specific biorecognition.

MIPs possessing dual functional monomers (methacrylic acid and 2-vinylpyridine) were synthesized by Thach et al. [10] to yield materials that can be used for selective solid phase extraction of ciprofloxacin. The batch adsorption experiments demonstrated that the obtained MIPs showed a high adsorption capacity and selectivity toward ciprofloxacin. High recovery values were obtained when aqueous solutions were used. The described analytical procedure allows the designing of new adsorbents with high adsorption capacity and good extraction performance for highly polar template molecules.

In their review article, Lusina and Cegłowski [11] explored the mechanisms that can be used for controlled drug release from MIP hydrogels for transdermal drug delivery. They discussed applications of thermo-responsive, pH-responsive, and dual/multiple-responsive MIP hydrogels.

The other review article, prepared by Liu et al. [12], describes computational simulation modeling methods and the theoretical optimization methods of various molecular simulation calculation software for MIP preparation. The review summarizes the progress in research on and application of MIPs prepared by computational simulations and computational software in the past two decades.

This Special Issue has brought together experts that have studied and explored various aspects of MIPs. I want to thank all researchers who have contributed to the production of this Special Issue of *Polymers*. In addition, I would like to express my gratitude to the Editorial Team who helped prepare the “Advance in Molecularly Imprinted Polymers” Special Issue.
